# Low-cost optical system for laser phacoemulsification of cataracts

**DOI:** 10.1117/1.BIOS.2.2.022304

**Published:** 2025-04-29

**Authors:** Mitchell Harrah, Abdul Mohaimen Safi, Sadhu Moka, Ramesh Ayyala, Ashwin B. Parthasarathy

**Affiliations:** a University of South Florida, Department of Medical Engineering, Tampa, Florida, United States; b University of South Florida, Department of Electrical Engineering, Tampa, Florida, United States; c University of South Florida, Department of Ophthalmology, Tampa, Florida, United States

**Keywords:** phacoemulsification, ablation, cataracts

## Abstract

**Significance:**

Laser-based phacoemulsification is the preferred treatment for surgical removal of cataracts in the eye. Conventional methods of laser phacoemulsification use expensive, high-powered lasers that use femtosecond pulses to soften and emulsify cataracts, resulting in low- to middle-income countries relying on methods that lead to longer recovery times and higher rates of infection and astigmatism.

**Aim:**

We introduce and demonstrate the potential of laser phacoemulsification using a millisecond pulsed laser that can be realized using a low-cost diode laser.

**Approach:**

We show proof of concept for this device using computer simulations in COMSOL and with a prototype instrument tested on different tissue-simulating phantoms.

**Results:**

Our experiments show that the millisecond pulsed laser liquifies cataract-like tissues only at the point of illumination.

**Conclusions:**

A low-cost device for phacoemulsification can potentially reduce the burden of cataract surgeries in low-resource settings.

Statement of Discovery This work utilizes millisecond near-infrared laser pulses to demonstrate the ablation of cataract tissues through the use of gelatin phantoms. This could lead to a better standard of care within low- to middle-income countries, instead of the current route of care of capsular extraction.

## 1 Introduction

Cataracts are an emerging public health crisis for a population that is rapidly aging. In the United States, the Centers for Disease Control and Prevention estimated that over 30 million patients were affected by cataracts in 2020.^1^ Cataracts occur when older lens fibers solidify to create an opaque solid at the optical center of the ocular lens that manifests symptoms such as blurry vision and astigmatism and can lead to blindness if untreated. This results in a massive loss in a patient’s quality of life, affecting their day-to-day activities and their ability to participate in many societal functions.^2^

The current standard of care for the treatment of cataracts is surgical removal via a small incision (1.5 to 2.6 mm) using phacoemulsification. Phacoemulsification is a method in which the eye’s internal lens is emulsified with an ultrasonic handpiece and aspirated from the eye with the help of an operating microscope. Aspirated fluids are replaced with irrigation of balanced salt solution to maintain the anterior chamber volume, followed by intraocular lens implantation. More recently, optical methods such as femtosecond lasers have been used to perform certain steps of cataract surgery, including astigmatic correction, entry into the eye, cutting of the capsular bag, and slight softening of the hard nucleus. Here, an Nd:YAG crystal is used to create femtosecond pulses at ∼1064  nm, which heats and emulsifies the cataract core. These modern approaches facilitate faster healing, requiring minimal sutures.

However, the phacoemulsification technology ($130K), operating microscope ($100K), and femtosecond laser ($500K) machines are expensive to set up and operate, making this approach unaffordable in developing countries, rural areas, or other low-resource settings.^3^ The cost of cataract surgery can range from $380 to $1000,^4^ which is nearly double the annual income of most rural and developing areas.^4^ Furthermore, a femtosecond laser can result in minimal softening of the hard nucleus, often resulting in the additional use of conventional ultrasonic energy to complete the liquification.^3^ These machines also take up almost two square meters of floor space, reducing their portability between surgical suites and medical centers.^3^ The lack of portability also creates multiple logistic hurdles that prospective patients must endure, such as requiring travel to the nearest medical center with the technology, finding an affordable place to stay during initial recovery and travel/stay for post-operative care.^5^ Unfortunately, the cataract burden is highest in the poorer sections of the population. The alternative to femtosecond phacoemulsification is extracapsular cataract extraction, which requires a large incision (5 to 7 mm) and closure with sutures, leading to a longer healing period.^3^^,^^6^ Critically, these approaches result in the occurrence of high rates of astigmatism, or blurry vision, after the surgery.^6^ Thus, there is a need for a low-cost, highly effective, safe surgical technology that is portable, cheap, and can deliver similar or better results to the current standard of phacoemulsification.

Here, we present a low-cost alternative for the lens fragmentation process of laser phacolysis that does not use a femtosecond laser for phacoemulsification. Our approach utilizes a high-powered pulsed laser diode, accompanied by a custom laser mount and a custom fiber specifically designed to fit within a surgical probe. In this paper, we demonstrate the feasibility of this approach with simulations and experiments on tissue-simulating phantoms. We first validate our approach using simulations of optical–thermal interactions with ocular tissues in COMSOL. Our experiments show that the laser delivery method will raise cataracts’ temperatures to a sufficient level to emulsify them, without damaging any surrounding tissues. Further, we validate our bench-top prototype on custom tissue-simulating phantoms *in vitro*. In gelatin-phantom models, we show that the heat generated by the laser is confined to a small region when applying millisecond pulses.

## 2 Materials and Methods

### 2.1 Operating Principle

The operating principle behind the proposed low-cost alternative for laser phacolysis is the utilization of millisecond pulses to achieve ablation of the cataract. Ablation could occur under continuous laser illumination, but the process is vastly inefficient and could cause thermal damage to the cornea in patients.^7^ Instead, pulsed laser illumination leverages thermal, mechanical, and chemical phenomena to create efficient and safe ablation.^7^ To achieve efficient ablation, the primary consideration is the pulse width duration in comparison with the thermal and mechanical critical relaxation times of tissue. When the pulse width of the laser is shorter than the thermal and mechanical critical relaxation times, the thermal energy and acoustic stress will be contained within the illumination volume.

The thermal relaxation time, $t_{t}$, is calculated as $t_{t}=\frac{1}{\kappa (3\mu_{a}(\mu_{a}+\mu_{s}^{\prime }))}$.^8^ Here, $\mu_{a}$ and $\mu_{s}^{\prime }$ are the absorption and reduced scattering coefficients of the cataract, respectively, and κ is the thermal diffusivity given by $\kappa =\frac{K}{C_{p}\rho }$, where K is the thermal conductivity, $C_{p}$ is the specific heat at constant pressure, and ρ is the density of the cataract.

Per mechanical considerations, the mechanical stress ($\sigma_{p}$) generated by the laser light for ablation is given by, $\sigma_{p}=A\Gamma \mu_{a}\Phi_{0}$, where A is the stress confinement factor, a measure of how efficiently the stress generated by the laser pulse is confined within the material, Γ is the Grüneisen coefficient, and $\Phi_{0}$ is the incident radiant exposure. The stress confinement factor (A) further depends on the laser pulse width ($t_{p}$) and the mechanical relaxation time ($t_{m}$) and can be calculated as

$$A=\frac{1-e^{-\frac{t_{p}}{t_{m}}}}{\frac{t_{p}}{t_{m}}},$$

with $t_{m}=\delta /v$, where v is the speed of sound in the material. The mechanical relaxation time is a measure of the dispersion of mechanical energy in the material. δ is the light penetration depth given by $\delta =\sqrt{\frac{1}{3\mu_{a}(\mu_{a}+\mu_{s}^{\prime })}}$.^7^

**Fig. 1:**
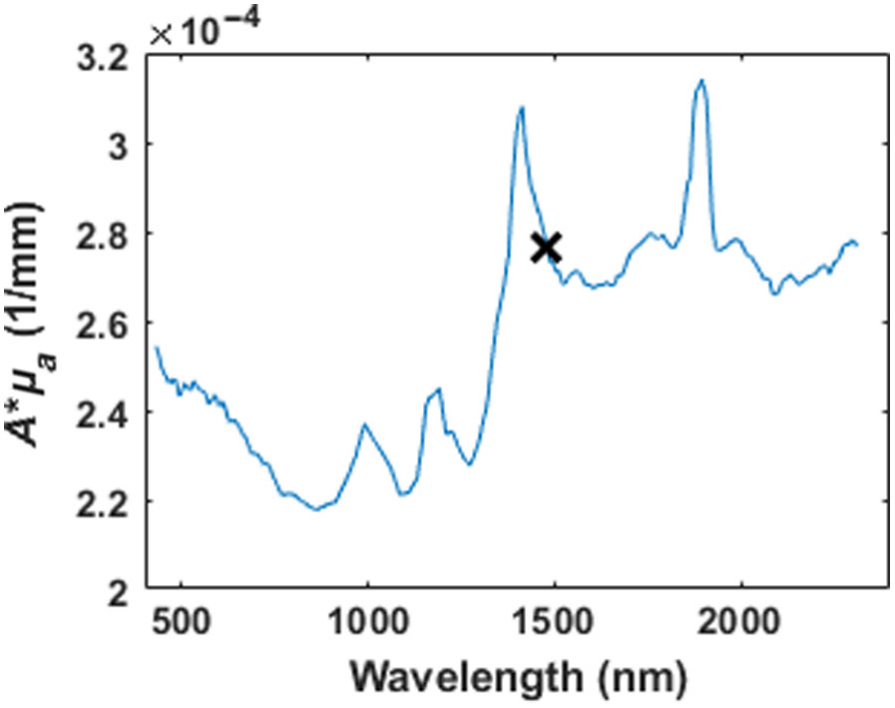
Plot of the ablation figure of merit $A\mu_{a}$ as a function of wavelength for 10-ms laser pulse width. “X” marks the figure of merit value for 1480 nm, which is used in this study.

It is important to recognize that $\mu_{a}$ and $\mu_{s}^{\prime }$ (and hence δ and A) all depend on the wavelength of light used to illuminate the tissue. To determine the best wavelength for laser ablation of cataracts, we calculated the product $A\mu_{a}$, a metric that combines the effects of stress confinement and tissue laser light absorption, both of which will increase the ablation stress. For this parameter, the goal is to find the maximum values of Aμ_a for diode wavelength selection. Figure 1 shows the ablation figure of merit for an operating pulse width of 10 ms, which leads us to a choice of 1480 nm based on available off-the-shelf high-power laser diodes. For more information, Fig. 7 in Appendix A plots $A\mu_{a}$ as a function of wavelength for different pulse width durations. Table 1 provides the values used to calculate the critical relaxation times of $t_{t}=5.67\;s$ and $t_{m}=2.86\times 10^{-7}\;s$ for 1480-nm laser illumination.

**Table 1:** Properties used to calculate the penetration depth and critical relaxation time.

Property (units)	Notation	Value
Density ($\mathrm{kg}/m^{3}$)	ρ	1050^9^
Thermal conductivity [W/(m*K)]	K	0.4^9^
Specific heat [J/(kg*K)]	$C_{p}$	3000^9^
Absorption coefficient at 1480 nm (1/cm)	$\mu_{a}$	9.67^10^
Reduced scattering coefficient at 1480 nm (1/cm)	$\mu_{s}^{\prime }$	9.03^10^
Penetration depth (cm)	Δ	0.03
Thermal diffusivity (${\mathrm{cm}}^{2}/s$)	κ	0.0013
Speed of sound (mm/s)	v	1,500,000^8^
Thermal critical relaxation time (s)	s	5.67
Mechanical critical relaxation time (s)	$t_{m}$	$2.86\times 10^{-7}$

### 2.2 Instrumentation

**Fig. 2:**
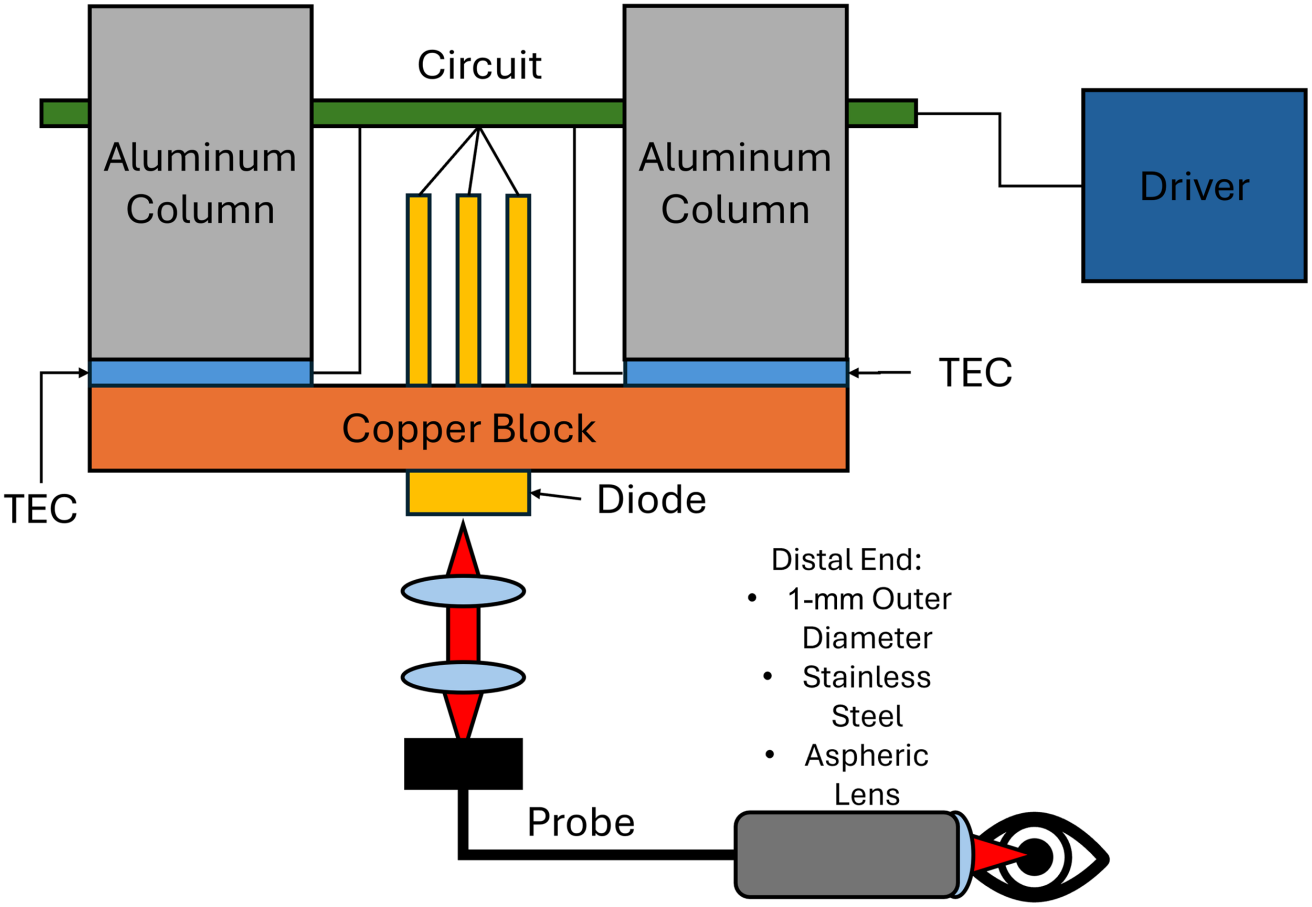
Diagram of the prototype used in the experiments. The electrical components were housed in an aluminum shell. A custom diode laser system was created to deliver the laser light directly to the target.

A custom low-cost laser phacolysis instrument was fabricated using a high-power pulsed laser diode system. The device features a 1480-nm, 2000-mW laser diode (L1480G1, Thorlabs, Newton, New Jersey, United States) attached to a custom laser mount, as shown in Fig. 2.

The laser diode rests on a copper block with two thermoelectric cooling (TEC) elements (Laird Thermal Systems, 430856-501, Rosenheim, Germany) attached to it. Two solid aluminum columns were attached to the copper block to facilitate cooling. In addition, each column has three thermal fins to help with heat dissipation. The entire arrangement was secured in a box with four high-speed fans. The laser and the TEC elements were connected to a custom printed circuit board, which is connected to an external laser diode controller (ITC4020, Thorlabs, Newton, New Jersey, United States). Through a voltage feedback circuit connected to the external laser diode controller, the optical output of the laser diode is stabilized, adding increased protection against over-driving the diode. The laser diode controller electronically pulses the laser at a user-defined frequency and duty cycle. Finally, the optical output of the laser diode was collimated and coupled to a custom fiber optic probe (Fiberoptic Systems Inc., Simi Valley, California, United States) with 1-mm bundled borosilicate fiber core (0.55 NA) for easy illumination of the sample. The proximal end of the fiber had a 1.5 mm diameter, 2 mm focal length, and 0.38 NA lens (Edmund Optics, Barrington, New Jersey, United States) to focus the laser light on a spot.

### 2.3 COMSOL Simulation

A simulation of optical–thermal interaction in the cataract/lens was performed using COMSOL to verify that the laser diode operating at millisecond pulses can cause phacolysis without damaging surrounding tissue. Briefly, the sclera of the eye was modeled as a perfect circle with a thickness of 0.96 mm. The shape of the cornea and lens was modeled using the equation, $r^{2}+(1+Q)z^{2}-2zR=0$, where r is the radial position on the curve, Q is the asphericity, z is the position on the z-axis, and R is the maximum radius of the curve.^11^ Here, we have ignored the contribution of the fovea. Further, the iris was simplified to a diamond shape with a maximum thickness of 0.46 mm.^12^ Finally, a three-dimensional model was generated, assuming the eye was axisymmetric (see Fig. 8 in Appendix B).^11^ Tables 7 and 8 (Appendix B) display geometric and thermal properties of the different eye tissue types.^13^ Here, we assume that the cataract fills the lens entirely. Tables 9 and 10 (Appendix B) provide the material properties of the aqueous humor and cataract, respectively^10^^,^^14^ at 1480 nm. The density of the aqueous humor was modeled as $\rho =\rho_{0}[1-\beta (T-T_{0})]$. Here, $\rho_{0}$ represents the reference density in $\mathrm{kg}/m^{3}$, β is the volume expansion coefficient in $K^{-1}$, and $T_{0}$ is the reference temperature in °C.^15^ Given that we are only interested in the thermal interactions on the cataract, we have selected to only define the solid material properties of the cataract and none of the other solid media in the ocular region.

The primary objectives of the COMSOL simulation were to measure the maximum temperatures and stress at the optical center of the lens and any tissue damage that occurs on the cornea for different pulse widths and optical powers. Optical, thermal, and mechanical effects of laser irradiation, with a spot size radius of 287  μm, on the cataract were performed for a duration of 10 s using the bioheat transfer, solid mechanics, radiative beam in absorbing medium, and laminar flow physics modules and the thermal expansion, heat transfer with radiative beam, and nonisothermal flow multiphysics modules. The simulation began with the laser being “on” for 10 s. The laser was applied in the negative z-direction, pulsing at the specified pulse width interval. After the 10 s, the simulation examined any effects that may occur 110 s immediately after the lasing process.

### 2.4 Tissue Simulating Phantom

**Fig. 3:**
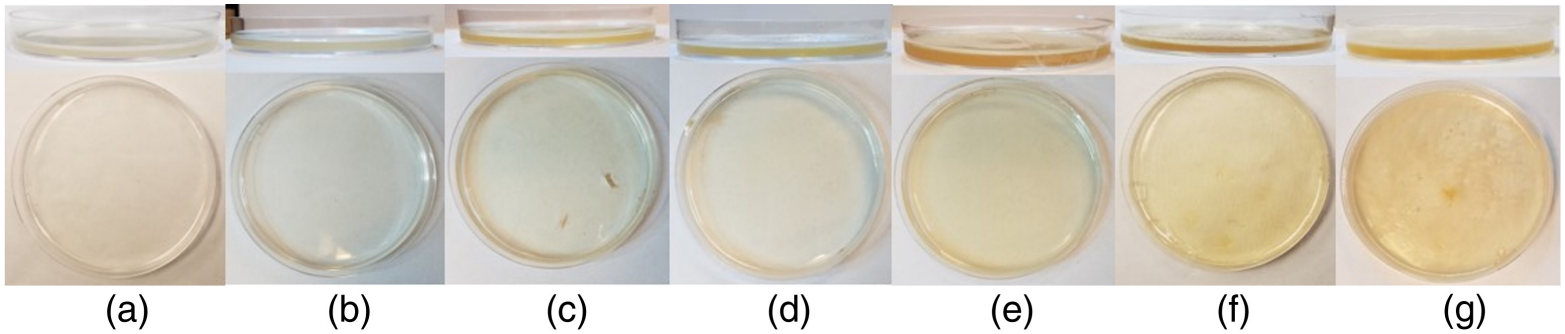
LOCSIII classification of gelatin cataract samples as rated by six independent ophthalmologists. The graph shows a sigmodal relationship between LOCSIII grades and gelatin concentration. Although the gelatin reached a large solubility, an average LOCSIII grade of 5 was not achieved.

Experimental validation of the laser system was performed using tissue-simulating gelatin phantoms. These phantoms have previously been used as a substitute for cataracts in optical coherence tomography (OCT)^16^ and ultrasound^17^ characterization studies; they mimic the mechanical/optical/thermal properties of the cataract. Multiple gelatin phantoms were fabricated to simulate different grades of cataracts using methods described elsewhere,^16^^,^^17^ with gelatin concentrations defined in Table 11 (Appendix C). Briefly, the desired mass of gelatin (Sigma-Aldrich, G6144, type A, 90 to 110 g bloom, St. Louis, Missouri, United States) was dispersed in 50 mL of distilled water that had been brought to a boil. Once the water began to boil rapidly, the gelatin powder was slowly added to the heated solution, whereas the solution underwent rigorous mixing to prevent the gelatin from forming large solid particles. After the mixing, the solution was poured into Petri dishes to a height of 5 mm, which matches the approximate thickness of ocular lenses.^18^ Seven such samples were fabricated and refrigerated for 24 h; pictures of each sample were taken for a survey to grade them on a standard cataract scale (Fig. 3).

### 2.5 Classification of Tissue Simulating Phantoms

**Fig. 4:**
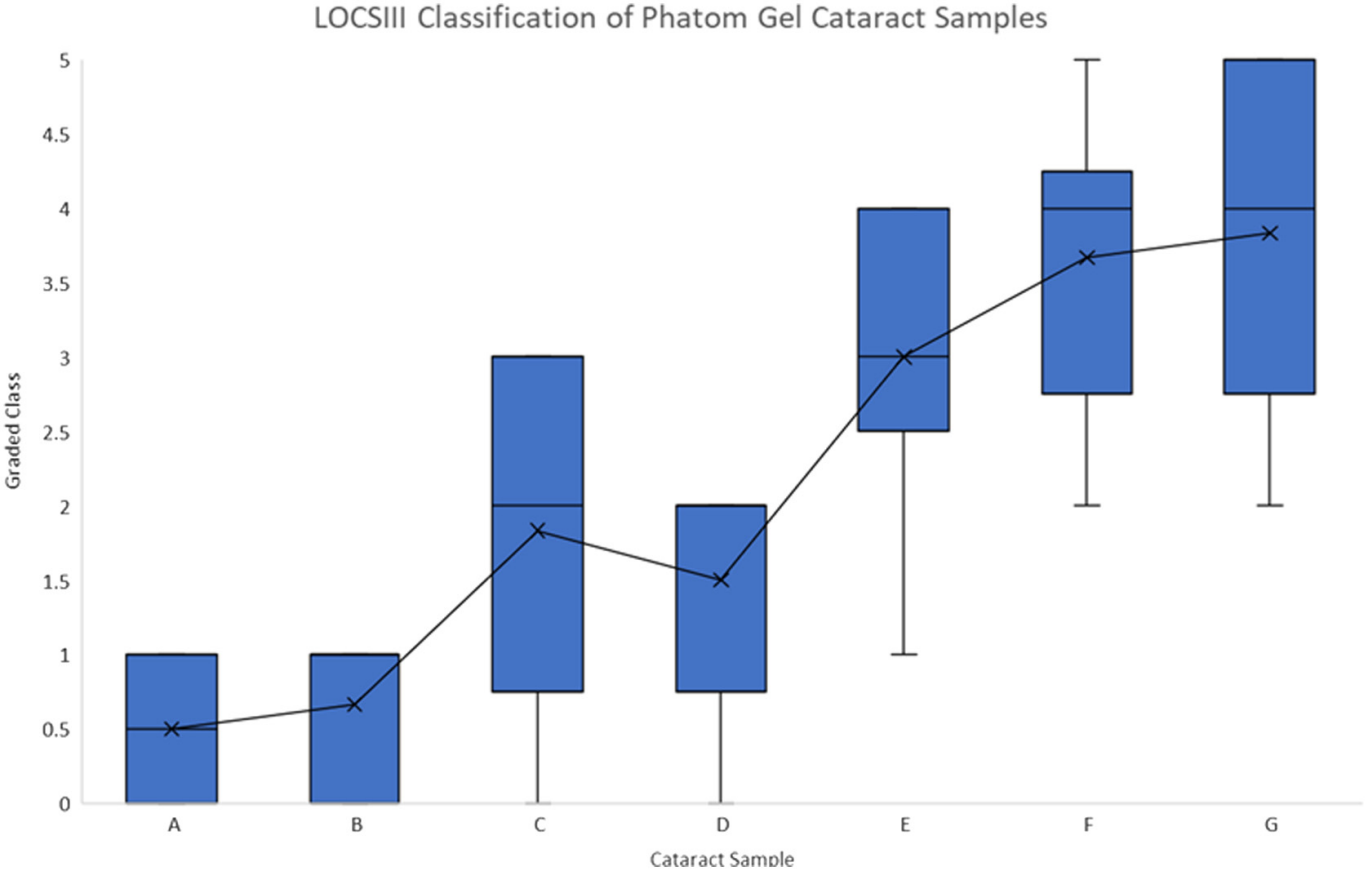
LOCSIII classification of gelatin cataract samples as rated by six independent ophthalmologists. The graph shows a sigmodal relationship between LOCSIII grades and gelatin concentration. Although the gelatin reached a large solubility, an average LOCSIII grade of 5 was not achieved.

Cataract phantoms were classified using the Lens Opacity Classification System III (LOCSIII).^4^ Six ophthalmologists individually rated seven gelatin phantoms according to LOCS III, based on randomly presented pictures of the phantoms’ top and side views. The result of this rating is presented in Fig. 4, showing a sigmoidal curve, which flattens out at around grade 4.

### 2.6 Experimental Methods

For the tissue phantom validation experiments, the laser diode system was used to illuminate the sample surface with 1 W, measured at the sample surface, maximum power, at 10-, 30-, 50-, 70-, and 90-ms pulse widths, each with a 50% duty cycle, as well as for continuous wave illumination. The pulse widths were selected to match those used in the COMSOL simulation. The laser was positioned to illuminate the surface of the gelatin phantom from a fixed height (approximately 10 mm) for up to a maximum of 5 min while the gelatin liquefied. A picture of the surface of the phantom was recorded using a digital camera. The area of the indentation was estimated using a custom algorithm implemented in MATLAB. This procedure was repeated five times for each phantom and pulse width. The Wilcoxon rank-sum test was used to determine if the resulting damage areas were statistically different for each phantom/pulse width.

## 3 Results

### 3.1 COMSOL Simulation Results

The primary goal of the COMSOL simulations was to determine the magnitude and extent of temperature increases in the lens due to laser irradiation. Table 2 presents the maximum temperature reached at the refractive center of the lens for various laser illumination powers and pulse widths. The radius of the laser spot size was fixed at 287  μm to match that of the optical probe. In general, higher irradiation powers cause larger temperature increases. Critically, for every irradiation power, pulsed operation results in lower temperature increases compared with continuous wave operation.

**Table 2:** Maximum temperatures at the refractive center of the human lens under various pulse widths and laser radius of 287  μm.

Power (mW)	Max temperature (°C) at					
	Continuous	10 ms	30 ms	50 ms	70 ms	90 ms
500	110.54	75.89	76.36	76.83	77.35	78.01
1000	182.17	111.49	112.40	113.40	114.46	115.60
1500	257.80	147.43	148.80	150.30	151.90	153.59
2000	343.17	183.53	185.34	187.26	189.37	192.00
2500	455.32	220.48	222.74	225.24	227.87	231.13
3000	636.95	259.03	261.72	264.68	267.83	271.80

Further, to evaluate the effect of the heat localization that we would expect from the ablation, we calculated the maximum area of the lens that reaches or exceeds 100°C (Table 3). As with Table 2, the results in Table 3 show that the area of the lens where temperature exceeds 100°C increases with laser power and decreases with pulse width; the latter result demonstrates the effect of thermal confinement.

**Table 3:** Area of the lens exceeding 100°C under various pulse widths and laser radius of 287  μm.

Power (mW)	Area (${\mathrm{mm}}^{2}$)					
	Continuous	10 ms	30 ms	50 ms	70 ms	90 ms
500	.73	0.13	0.15	0.16	0.18	0.19
1000	1.90	0.78	0.78	0.79	0.79	0.80
1500	2.89	1.37	1.37	1.38	1.38	1.39
2000	3.74	1.91	1.92	1.92	1.93	1.93
2500	4.56	2.42	2.42	2.43	2.43	2.44
3000	5.36	2.89	2.89	2.89	2.90	2.91

Further, even with the lowered maximum temperatures, the tissue damage on the posterior corneal lens is a key point of interest. Table 4 shows whether any tissue damage was seen on the posterior corneal lens boundary. Tissue damage was defined to have occurred if the temperature reached 59°C for 60 s or if the peak temperature was 65°C.^19^ The results from Table 4 show that pulsed operation resulted in lower tissue damage to the posterior corneal surface.

**Table 4:** Temperature at the posterior corneal surface of the lens under various pulse widths and laser radius of 287  μm.

Power (mW)	Temperature (°C)					
	Continuous	10 ms	30 ms	50 ms	70 ms	90 ms
500	48.8	42.8	42.8	42.8	42.8	42.8
1000	60.93^a^	48.92	48.93	48.92	48.95	48.96
1500	73.09^a^	55.01	55.02	55.00	55.05	55.07
2000	84.89^a^	61.03^a^	61.05^a^	61.05^a^	61.10^a^	61.13^a^
2500	96.82^a^	67.02^a^	67.06^a^	67.05^a^	67.11^a^	67.17^a^
3000	108.97^a^	73.11^a^	73.02^a^	73.06^a^	73.12^a^	73.25^a^

a If corneal damage was present.

### 3.2 Tissue Phantom Experiment Results on Phantoms with LOCSIII Grades Less Than 2.5

**Fig. 5:**
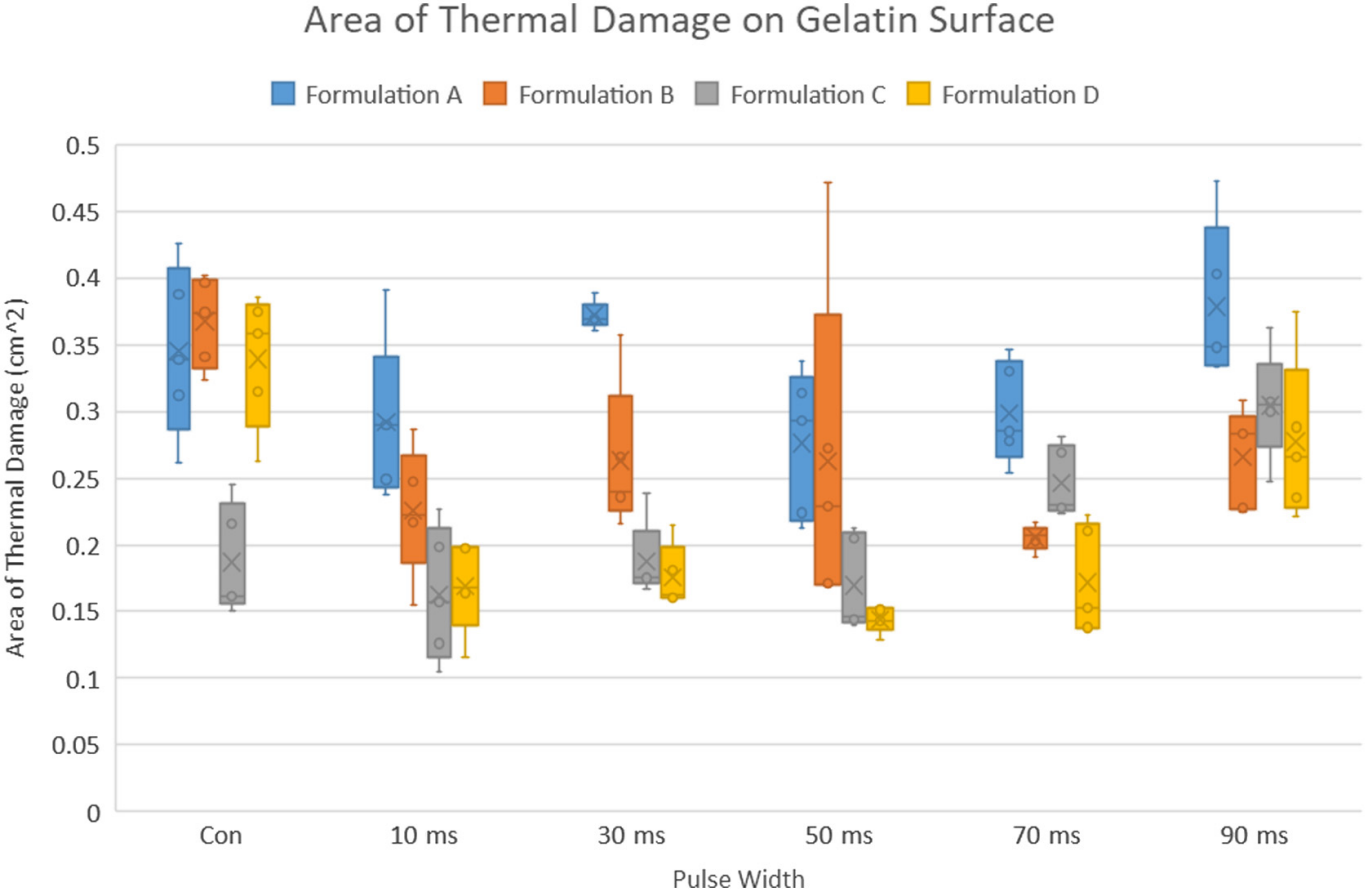
Area of thermal damage on less opaque gelatin phantoms (LOCSIII grade<2.5). For most formulations, there is no clear trend on the area of thermal damage. Formulation D was found to have some significantly different (lower) areas of thermal damage with the pulsed laser.

Figure 5 plots the area of thermal damage on the surface of the tissue (area of liquefaction) for sample formulations A through D for different pulse widths. These formulations comprise gelatin phantoms that were graded to be <2.5 on the LOCSIII scale. The ablative effect of laser on these samples does not present a clear relationship, but some trends are apparent. In most phantoms, the damage due to pulsed illumination was lower than those for continuous illumination. Table 5 shows the mean damage area for these phantoms. However, due to the fragile nature of these formulations (i.e., low levels of gelatin), the samples melted at relatively low temperatures and with minimal contact with the probe. It is possible that, due to the lower melting point of these mixtures, apparent features were not visible, which made calculating damage areas with the MATLAB algorithm prone to error.

**Table 5:** Median area (${\mathrm{cm}}^{2}$) of induced thermal damage on phantoms with LOCSIII grades less than 2.5.

Pulse width	A	B	C	D
Continuous	0.339	0.374	0.161	0.358
10 ms	0.29	0.222	0.157	0.168^a^
30 ms	0.369	0.24	0.176	0.162^a^
50 ms	0.293	0.229	0.146	0.143^a^
70 ms	0.285	0.207	0.23	0.153^a^
90 ms	0.348	0.283	0.305	0.266

a Statistically significant difference compared with continuous.

### 3.3 Tissue Phantom Experiment Results on Phantoms with LOCSIII Grades More Than 2.5

**Fig. 6:**
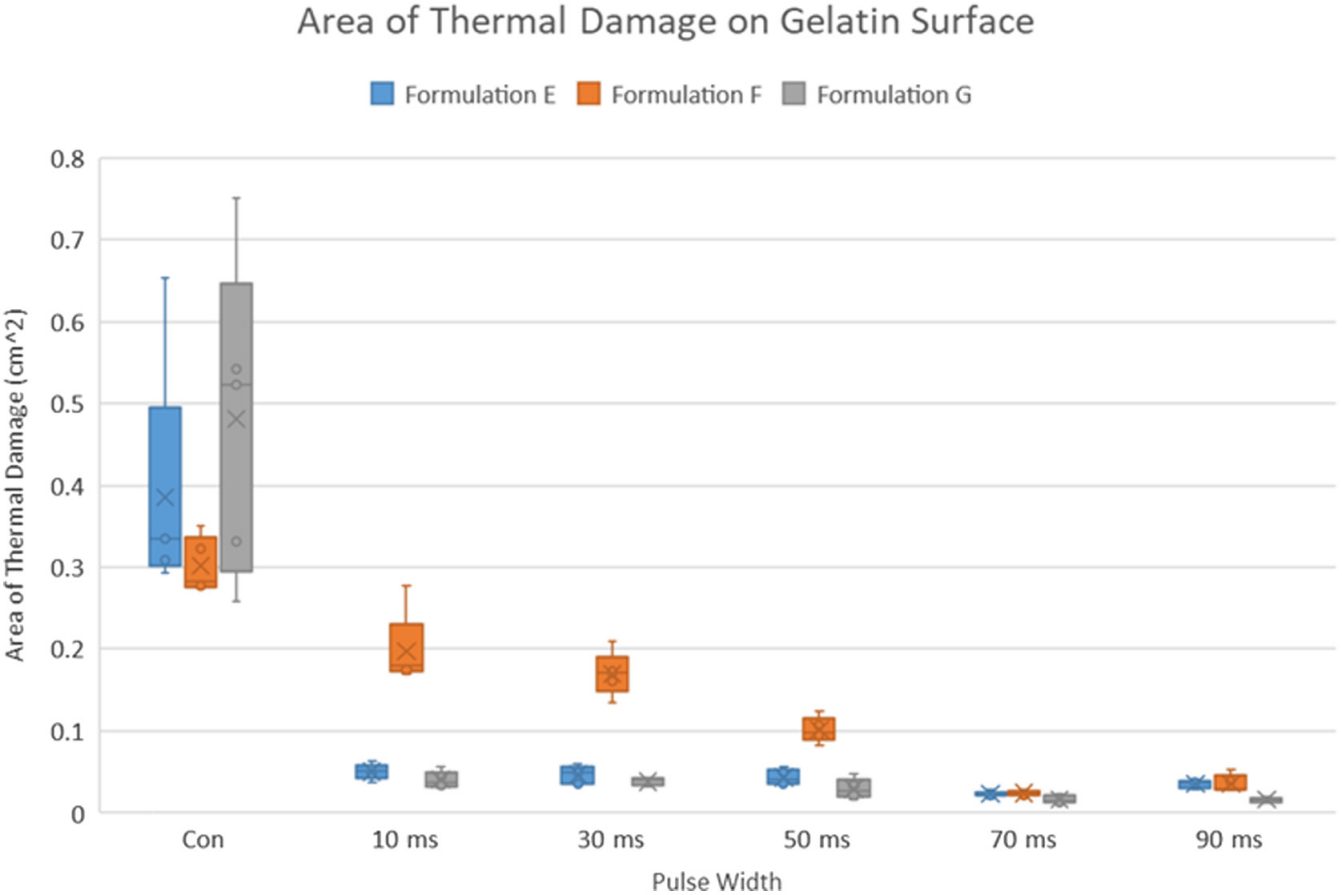
Area of thermal damage on more opaque gelatin phantoms (LOCSIII grade>2.5). These samples show a significant difference between the pulsed laser application and the continuous laser operation.

Figure 6 and Table 6 show the damage areas recorded for the three highest-graded formulations of gelatin mixtures (formulations E through G), which represent cataracts graded above 2.5 on the LOCSIII scale. In all phantoms, the damaged area for continuous illumination was greater than for pulsed illumination, with strong statistical significance. Interestingly, the effects did not monotonically increase with phantom grade. The effects on formulation F were weaker than those on formulation G.

**Table 6:** Median area (${\mathrm{cm}}^{2}$) of induced thermal damage on phantoms with LOCSIII grades more than 2.5.

	E	F	G
Continuous	0.335	0.283	0.523
10 ms	0.051^a^	0.18^a^	0.037^a^
30 ms	0.049^a^	0.171^a^	0.041^a^
50 ms	0.04^*^	0.098^a^	0.027^a^
70 ms	0.022^a^	0.024^a^	0.014^a^
90 ms	0.037^a^	0.029^a^	0.016^a^

a Statistically significant difference compared with continuous.

## 4 Discussion

In this contribution, we present our initial experience with using a custom, low-cost laser device for the phacoemulsification of cataracts. A key feature of this device is its use of millisecond pulses to achieve laser phacoemulsification; millisecond pulsed lasers can be realized at a fraction of the cost of conventional femtosecond lasers. These preliminary experiments aim to establish that millisecond laser pulses can produce localized heating of the cataract, leading to phacolysis. For successful thermal phacolysis, the laser pulse width needs to be smaller than the thermal relaxation time (5.67 s) of the tissue. This ensures that the heat generated by the laser is confined to the illumination spot and is not lost by thermal diffusion. Our results, obtained from both COMSOL simulations and tissue phantom experiments, confirm that millisecond laser ablation can achieve thermal confinement for cataract-like tissues.

Finite element simulations of heat transfer in the eye confirmed that the millisecond pulse laser could successfully increase the temperature in the cataract while maintaining minimal thermal damage to other regions of the eye. Although we observe a significant rise in temperature within the ocular lens, the COMSOL model indicates that the surrounding tissues, such as the posterior corneal tissues, will remain undamaged due to the convective fluid flow of the aqueous humor. As expected, these effects are dependent on the power of the laser. These simulations also demonstrated that pulsed operation of the laser resulted in temperature increases in smaller areas of the cataract compared with continuous wave operation. Given the larger pulse widths and probe size, we observed a higher temperature increase than published data seen in other femtosecond research.^20^ It should be noted, however, that our measurement is taken exactly at the point where the laser is applied, compared with other works that use different reference points (e.g., lens surface).^16^^,^^21^

We note a few limitations of the simulations. First, the simulation does not account for the movement of the probe around the optical lens, which may distribute the thermal effects over a larger area. Second, the entire lens is treated as a solid cataract, whereas 80% of the cataract consists of a central hard portion called the nucleus, which is surrounded by an outer soft shell called the cortex. Third, based on COMSOL’s current ability to analyze ablation, the simulation does not feature a moving boundary based on the real-world changes seen in a current surgery. Fourth, we do not directly simulate the effects of the laser on retinal tissues. The divergence of the laser illumination coupled with high light absorption in the vitreous humor suggests that irradiance levels at the retina will be low. Further studies are required to estimate/evaluate this. Finally, the simulation does not account for phase changes that may occur during surgery due to the ablation process, such as the creation of a slurry-like solution inside the ocular lens or the liquid material in the lens briefly becoming plasma. Nevertheless, we note that these limitations did not prevent us from examining the fundamental question posed by this work, i.e., can millisecond pulses cause phacoemulsification?

**Fig. 7:**
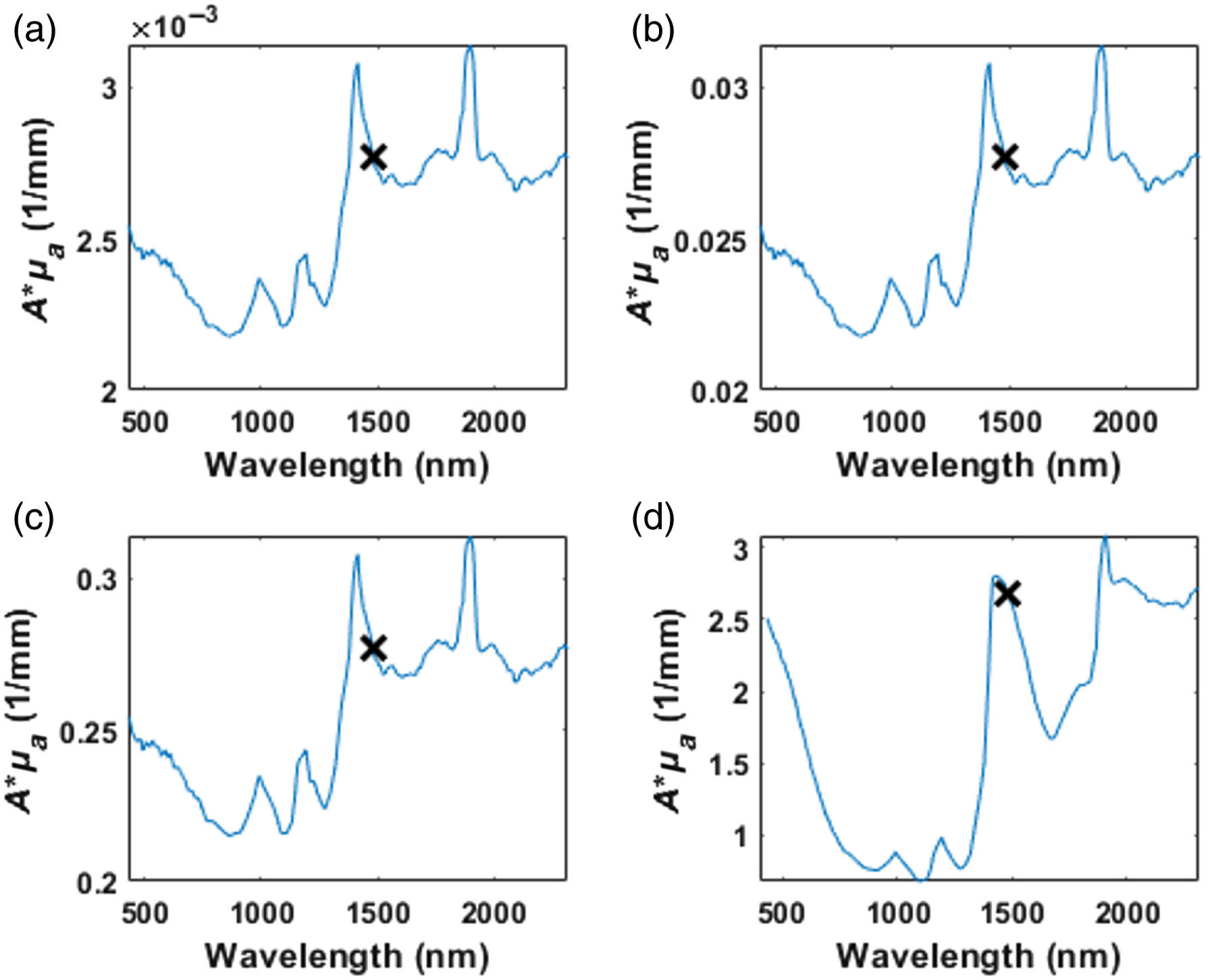
Effects of stress confinement and absorption as calculated by the metric $A\mu_{a}$ as a function of wavelength, for different pulse widths (a) 1000  μs, (b) 100  μs, (c) 10  μs, and (d) 1  μs.

In addition to the simulations, we successfully constructed a prototype millisecond pulsed laser diode system for phacolysis and tested it on cataract-simulating gelatin samples. Critically, we fabricated multiple samples and were able to categorize them according to the clinical grades of cataracts seen in the operating room. Here, we showed that pulsed operation of the laser was able to constrain the thermal effects to a smaller area compared with the continuous operation of the laser. By constraining the thermal effects to a small area, we created a fragmentation pattern seen in other clinical testing of phacoemulsification.^20^ We note here that our pulse widths are much higher than the mechanical relaxation time of the cataracts ($2.86\times 10^{-7}\;s$), leading us to speculate that our ablative process is primarily thermal. Nevertheless, we do induce mechanical stresses in the tissue, which help with the ablative process. Clearly, reducing the pulse width to a value that is closer to the mechanical relaxation time will amplify the mechanical effects (see Fig. 7, Appendix A).

Although these preliminary experiments demonstrate feasibility, we note that we used a rather simple tissue phantom. For example, Mencucci et al.^20^ used a commercially available laser calibration gelatin cylinder (Bausch + Lomb, Rochester, New York, United States) in their experiments. This phantom presents a more realistic geometry for testing and is commonly used in operating rooms to calibrate femtosecond lasers. Such realistic phantoms could account for model temperature dissipation in the eye due to motion and blood flow. Furthermore, our phantom experiments quantified the thermal effects by measuring the liquefaction area rather than temperature increase; the latter could have been accomplished with a temperature sensor to directly compare the phantom results to those from COMSOL.^20^ Validation in these realistic tissue phantoms is an important next step. Finally, the simulation and bench-top experiments used laser pulses with a 50% duty cycle. This allowed us to compare the effects of laser pulsing with the same average laser power across different pulse durations and helped ensure that the thermal cooling duration was not greater than the illumination duration. More tests are necessary to examine the effects of cooling durations that are longer than the illumination duration, i.e., with different duty cycles. These bench-top tests offer validation that the millisecond pulsed diode laser holds the potential for low-cost phacoemulsification of cataracts, which could yield significant results in cataract care in regions of the world where capsular extraction is prevalent. Important future steps include extending these experiments to *ex vivo* samples of bovine or pig eyes.

## 5 Appendix A: Figure Displaying the Effects of Pulse Length Across Wavelengths

We modeled the effects of stress confinement and absorption as an efficiency coefficient. Figure 7 plots the change in the efficiency coefficient as a function of illumination wavelength for different pulse widths.

## 6 Appendix B: Tables and Figures for COMSOL Simulation Setup

**Fig. 8:**
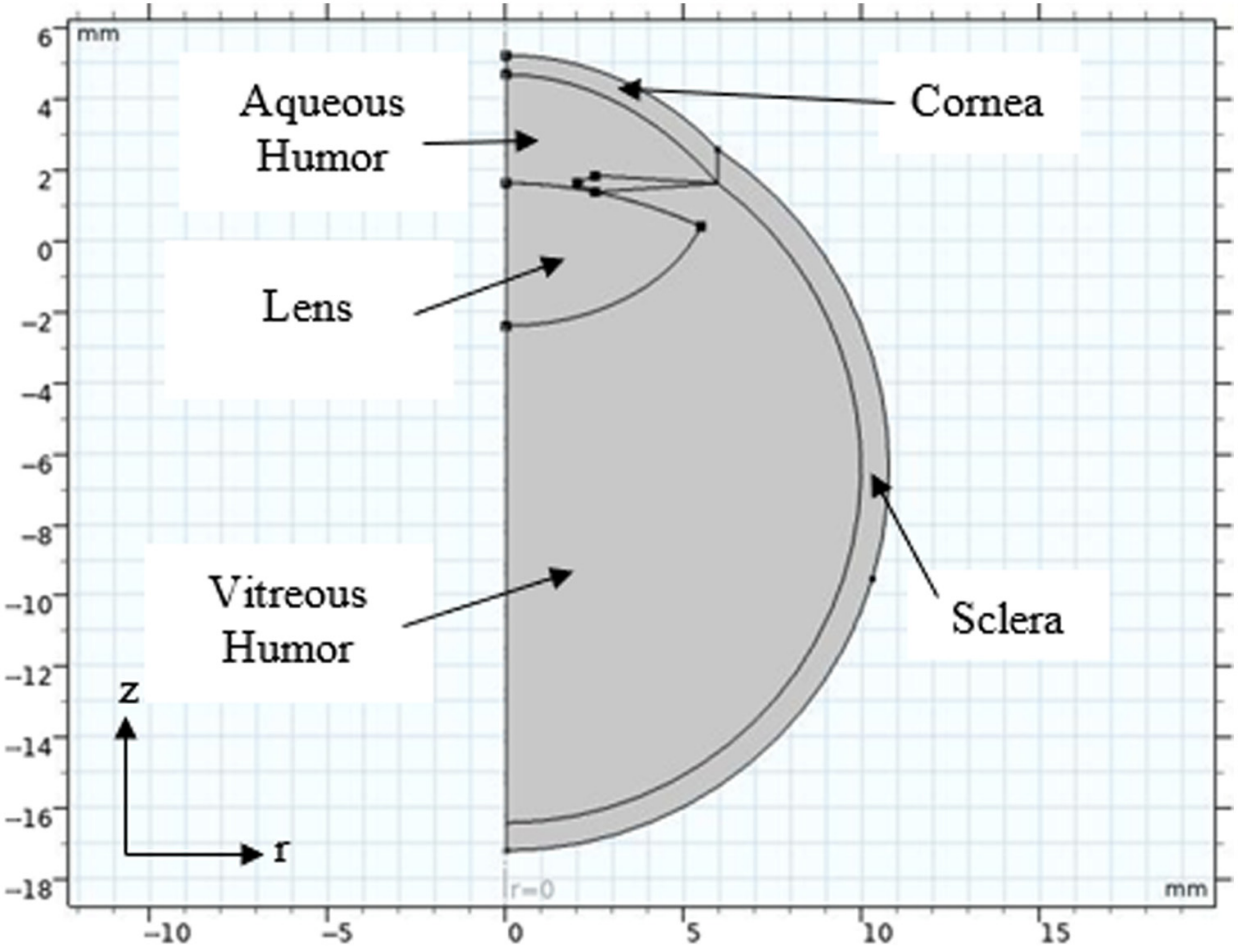
Final geometry of the ocular region.

This appendix describes the parameters used to create the various geometries for the eye model within COMSOL. Table 7 describes the complex geometry of the cornea. Figure 8 displays the final simulation geometry. Tables 8–10 describe the properties of the ocular biological material.

**Table 7:** Properties used to simulate ocular structure (positive or negative orientation denotes if the curve is concave or convex).^11^

Curve	Asphericity	Radius (mm)	Apex location (mm)	Orientation
Anterior lens	−0.94	12.40	1.59	Negative
Posterior lens	0.96	8.10	−2.59	Positive
Anterior cornea	−0.18	7.77	5.19	Negative
Posterior cornea	−0.60	6.40	4.64	Negative

**Table 8:** Selected properties of ocular biological materials.^22^

Material	Density ($\mathrm{kg}/m^{3}$)	Thermal conductivity [W/(m*K)]	Heat capacity at constant pressure [J/(kg*K)]
Cornea	1050	0.58	4178
Aqueous humor	$\rho =\rho_{0}[1-\beta (T-T_{0})]$	0.58	3997
Lens	1050	0.40	3000
Vitreous humor	1050	0.603	4178
Sclera	1000	1.0042	3180
Iris	1050	1.0042	3180

**Table 9:** Aqueous humor material property values.^15^

Property (units)	Value
Reference density ($\mathrm{kg}/m^{3}$)	996
Volume expansion coefficient ($K^{-1}$)	0.0003
Reference temperature (°C)	34
Dynamic viscosity ($N*s/m^{2}$)	0.00074
Specific heat ratio (1)	1.66

**Table 10:** Cataract lens material property values.

Property (units)	Value
Absorption coefficient (${\mathrm{cm}}^{-1}$)	9.67^10^
Young’s modulus (kPa)	6000^14^
Poisson’s ratio	0.47^14^
Volume expansion coefficient ($K^{-1}$)	0.0003^14^

## 7 Appendix C: Concentration of Gelatin Used in Different Tissue Phantom

Table 11 displays the concentrations of gelatin used to create the phantom cataract tissues.

**Table 11:** Formulations for cataract phantoms.

Formulation label	Mass of gelatin per 50 mL of water (g)
A	1.67
B	3.33
C	7.40
D	11.10
E	14.80
F	18.50
G	22.20

## Data Availability

All data in support of the findings of this paper are available within the article or as supplementary material.
